# Neuroprotective Effect of Methanolic Ajwa Seed Extract on Lipopolysaccharide-Induced Memory Dysfunction and Neuroinflammation: In Vivo, Molecular Docking and Dynamics Studies

**DOI:** 10.3390/plants12040934

**Published:** 2023-02-18

**Authors:** Vasudevan Mani, Minhajul Arfeen, Devendra Kumar Dhaked, Hamdoon A. Mohammed, Palanisamy Amirthalingam, Hossam A. Elsisi

**Affiliations:** 1 Department of Pharmacology and Toxicology, College of Pharmacy, Qassim University, Buraydah 51452, Saudi Arabia; 2 Department of Medicinal Chemistry and Pharmacognosy, College of Pharmacy, Qassim University, Buraydah 51452, Saudi Arabia; 3 Department of Pharmacoinformatics, National Institute of Pharmaceutical Education and Research (NIPER)-Kolkata, Kolkata 700054, India; 4 Department of Pharmacognosy and Medicinal Plants, Faculty of Pharmacy, Al-Azhar University, Cairo 11371, Egypt; 5 Department of Pharmacy Practice, Faculty of Pharmacy, University of Tabuk, Tabuk 47512, Saudi Arabia; 6 Department of Clinical Pharmacology, Faculty of Medicine, Zagazig University, Zagazig 44519, Egypt

**Keywords:** *Phoenix dactylifera*, cognitive deficits, cholinergic neurons, cyclooxygenase-2, cytokines, molecular modelling

## Abstract

Islamic literature has indicated that daily consumption of Ajwa dates heals a variety of chronic diseases and disorders. The current research investigates the neuroprotective effect of methanolic Ajwa seed extract (MASE) on lipopolysaccharide (LPS)-induced cognitive deficits using multiple approaches. For animal studies, MASE (200 and 400 mg/kg, p.o.) was administrated for thirty consecutive days, and four doses of LPS (250 µg/kg, i.p.) were injected to induce neurotoxicity. Memory functions were evaluated using elevated plus-maze and novel object recognition tests. Acetylcholine (ACh) and neuroinflammatory markers (cyclooxygenase (COX)-2, tumor necrosis factor (TNF)-α, interleukin (IL)-6, IL-10, and transforming growth factor (TGF)-β1) were estimated in brain tissues. Studies of molecular docking and dynamics were conducted to provide insight into the molecular-level mechanisms. MASE administration resulted in a significant reversal of LPS-induced memory impairment in both maze models. Both doses of MASE elevated the ACh levels in an LPS-treated rat brain. In addition, the extract lowered COX-2 and proinflammatory cytokines (TNF-α and IL-6) while increasing anti-inflammatory cytokines (IL-10 and TGF-β1) in LPS-treated brain tissues. Molecular modeling results revealed that the compound’s ellagic acid, epicatechin, catechin, kaempferol, quercetin, and apigenin have the potential to act as a dual inhibitor of acetylcholinesterase (AChE) and COX-2 and can be responsible for the improvement of both cholinergic and inflammatory conditions, while the cinnamic acid, hesperidin, hesperetin, narengin, and rutin compounds are responsible only for the improvement of cholinergic transmission. The above compounds acted by interacting with the key residues Trp84, Asp72, Gly118, Ser200, Tyr334, and His440, which are responsible for the hydrolysis of ACh in AChE, while the COX-2 is inhibited by interacting with the residues (Val349, Leu352, Tyr355, Tyr385, Ala527, Ser530, and Leu531) of the hydrophobic channel. By promoting cholinergic activity and protecting neuroinflammation in the rat brain, MASE provides neuroprotection against LPS-induced cognitive deficits. Our preliminary findings will help with further drug discovery processes related to neuroinflammation-related neurodegeneration.

## 1. Introduction

Inflammation in the brain causes cognitive impairments and neurodegenerative illnesses, such as Alzheimer’s disease (AD). Experimental studies have shown that the inflammatory process in the neuron causes cell death and neurodegeneration [1]. AD is a major cause of dementia, which is characterized by an inability to form new memories due to a malfunction of the episodic memory system. Currently, the prevalence of AD-associated dementia is projected to be approximately 40–50 million cases, and it is estimated to reach 74.7 million cases by 2030 [2]. 

Two major pathogenic alterations in AD are the abnormal formation of intracellular neurofibrillary tangles and the higher deposition of amyloid plaques. Furthermore, inflammatory causes and cholinergic dysfunctions are other important factors in AD and other neurodegenerative diseases [3]. Glial cells, such as microglia, in particular, provide primary immune surveillance in the CNS, and their activation causes oxidative stress and pro-inflammatory responses. When cytokines and chemokines are released, cyclooxygenases and phospholipase A2 are activated, causing inflammatory processes in neurons [4]. Furthermore, the loss of cholinergic neurons has been linked to neurodegenerative illnesses and other age-related cognitive deficits. Inflammation vulnerability and cholinergic deficiency in neurons are considered common symptoms of AD. In addition, neuroinflammation has been proposed as a major trigger for the accumulation of pathologically processed amyloid-β and its associated cholinergic neuron degeneration, as well as memory impairment [5].

A potent endotoxin lipopolysaccharide (LPS), produced from the outer cell membrane of gram-negative bacteria, causes cognitive and long-term behavioral deficits. The etiology of LPS-related memory impairments is connected to the causes of neuroinflammation, which is described by microglial activation and the production of numerous inflammatory mediators in the brain, including cytokines like IL-1β, TNF-α, and IL-6, as well as the triggering of the nuclear factor kappa B (NF-κB) system [6]. The degeneration of neuron populations, notably central cholinergic neurons, and synaptic plasticity impairment are also connected to LPS-induced memory deficits [7]. Using the results of variations in oxidative phosphorylation, oxidative stress, and mitochondrial dysfunctions, previous studies have shown that LPS enhances the production of neuroinflammatory markers [8,9].

Among a variety of date palms (*Phoenix dactylifera* L.; family Arecaceae), Ajwa is a typical fruit crop in Saudi Arabia from the Al-Madinah area. It is reported to be unique because of its nutritional as well as medicinal properties. Ajwa dates are high in dietary fiber, carbohydrates, vitamins, proteins, minerals, and lipids and are believed to be a good source of energy [10]. According to the literature, date fruits and seeds are used in the treatment of throat disease, atherosclerosis, pulmonary disease, mouth hygiene, and asthenia as an expectorant [11]. The phytochemicals in Ajwa date fruit, such as polyphenols and flavonoids, help lower cholesterol and prevent various cancers, diabetes, and cardiovascular diseases [10,12]. The pharmacological activities of Ajwa dates are further extended with antimicrobial activities comprising antibacterial, antiviral, antifungal, anti-inflammatory, nephrotoxic, and hepatoprotective activities [10,13]. In streptozotocin-induced experimental diabetic rats, an aqueous extract from Ajwa seed at a dose of 100 mg/kg resulted in lower blood glucose levels. Furthermore, in the same model, long-term administration of Ajwa seed extract was shown to enhance liver and kidney function while also balancing oxidative stress [14]. Treatment with Ajwa seed extract reduced blood cholesterol levels in a high-fat diet-induced hyperlipidemic rat model and had a hepatoprotective impact by lowering serum liver enzyme levels when combined with atorvastatin [15]. The targeting effect related to CNS, the administration of date seed extract in male rats, was found to save cortical neurons from cerebral-ischemia-related damage, and this mechanism was supported by the antioxidant capabilities of the extract [16]. In an AD transgenic mouse model, dietary supplementation with dates resulted in a significant reversal of memory loss by reducing plasma Aβ (Aβ42 and Aβ40) protein levels. Furthermore, using AD mice, the same therapy improved motor coordination and anxiety-related behavior [17]. According to a recent article from our laboratory, aqueous Ajwa seed extract at 200 and 400 mg/kg improved cognitive capacities in type-2 diabetic-induced rats by increasing CNS cholinergic activity [18]. The work was extended to show that altering the levels of both pro-inflammatory and anti-inflammatory cytokines in a rat brain could reverse the neuroinflammation caused by type 2 diabetes [19].

However, there is limited evidence related to the effects of Ajwa date seed on CNS disorders, particularly in terms of neuroprotection. Our objectives were to study the effect of methanolic Ajwa seed extract (MASE) on the enhancement of memory deficits in an LPS-induced rat model, and to explore the impacts of MASE on central cholinergic activity and specific neuroinflammatory markers in LPS-induced rats.

## 2. Results

### 2.1. Quantitate Analysis of Ajwa Extract

The quantitative assessments of the total phenolic contents in the MASE, including the polyphenols, flavonoids, and tannins, showed that these categories of secondary metabolites were present in significant amounts (Table 1). The measurements revealed the presence of a high quantity of the total phenolics measured at 76.06 mg GAE compared to the reported polyphenols in the data extract [20]. The findings also revealed the presence of a considerable amount of flavonoids and tannin contents, which were measured at the levels of 8.66 QE and 11.48 CE per gram of the extract, respectively. Plants rich in phenolics and flavonoids have been studied for their antioxidant and anti-inflammatory effects owing to the presence of these constituents [21,22,23]. These categories of secondary metabolites in edible fruits and greens have also been studied for their benefits in the prevention and treatment of neuroinflammation [24,25].

### 2.2. Acute Toxicity Study

There was no observation of toxic signs or animal death at the amounts of extract given orally, with the highest dose being 2000 mg/kg. Two lower MASE dosages (200 and 400 mg/kg, p.o.) were chosen for further memory study and ELISA analysis [18].

### 2.3. MASE Improved the Spatial Memory of LPS-Induced Rats in the Elevated Plus-Maze (EPM) Test

**Figure 1:**
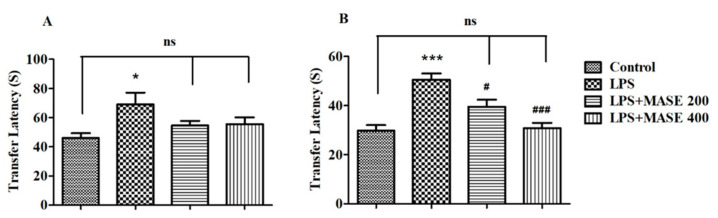
Effect of methanolic Ajwa seed extract (MASE) on (**A**) day 1 and (**B**) day 2 transfer latency of lipopolysaccharides (LPS)-induced rats using an elevated plus maze. The results are expressed by mean ± SEM (*n* = 6). One-way ANOVA [F(3,20) = 3.381, *p* < 0.05 for day 1 and F(3,20) = 15.03, *p* < 0.001 for day 2 transfer latency] followed by the Tukey–Kramer multiple comparisons test. * *p* < 0.05, and *** *p* < 0.001 as compared to the control group; ns—not significant as compared to the control group; ^#^ *p* < 0.05 and ^###^ *p* < 0.001 as compared to the LPS-induced group.

Figure 1 reveals the effect of MASE on transfer latency (TL) of LPS-induced cognitive deficiency in rats, analyzed using the EPM test. A one-way ANOVA demonstrated the significant effects of MASE on the retention TL values of day 2 [F(3,20) = 15.03, *p* < 0.001]. Further post-hoc analysis showed that four intraperitoneal injections of LPS extensively prolonged the TL values on day 1 (*p* < 0.05) and day 2 (*p* < 0.001) related to the control, indicating the impairment of spatial memory in LPS-induced rats. Oral administration of MASE significantly shortened the TL values of rats on day 2 at 200 mg/kg (*p* < 0.05) and 400 mg/kg (*p* < 0.001) when associated with LPS-induced rats. There was no alteration in day 1 TL values of MASE-treated animals as matched to the LPS-induced group.

### 2.4. MASE Enhanced the Cognitive Performance of LPS-Induced Rats in the Novel Object Recognition (NOR) Test

**Figure 2:**
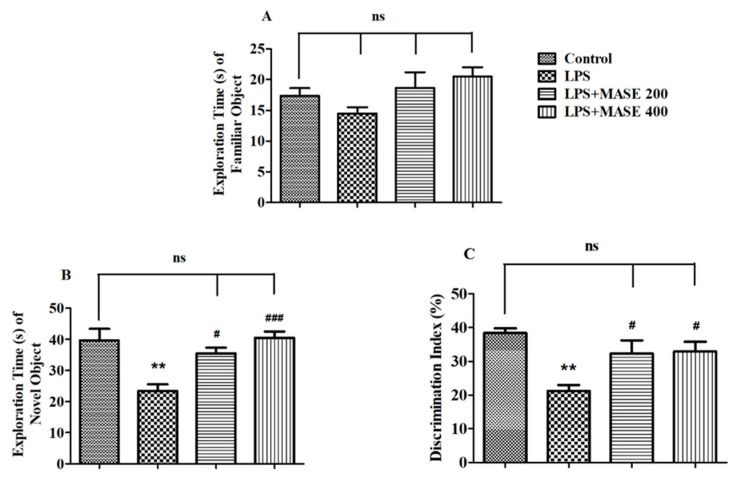
Effect of methanolic Ajwa seed extract (MASE) on (**A**) exploration time of a familiar object (FO) during the test session, (**B**) exploration time of a novel (NO) object during the test session, (**C**) discrimination index between two objects of LPS-induced rats using novel object recognition test. The results are expressed by mean ± SEM (*n* = 6). One-way ANOVA [F(3,20) = 2.254, *p* > 0.05 for exploration time of FO, F(3,20) = 9.603, *p* < 0.001 for exploration time of NO and F(3,20) = 7.097, *p* < 0.01 for discrimination index] followed by the Tukey–Kramer multiple comparisons test for comparisons within the groups. ** *p* < 0.01 as compared to the control group; ns—not significant as compared to the control group; ^#^ *p* < 0.05 and ^###^ *p* < 0.001 as compared to the LPS-induced group.

Figure 2 illustrates the effects of MASE on the studied behavioral parameters using the NOR test. In the test session, the results revealed the significant effects of MASE on the exploration time of a novel (NO) object [F(3,20) = 9.603, *p* < 0.001] and the discrimination index (DI) between two objects [F(3,20) = 7.097, *p* < 0.01] by employing a one-way ANOVA study. Extending the comparison between the designated groups revealed a considerably (*p* < 0.01) lower exploration time of NO as well as DI values as related to control. However, thirty days of treatment with MASE considerably improved the exploration time of NO (*p* < 0.05 with 200 mg/kg and *p* < 0.001 with 400 mg/kg) by rats when compared with LPS-induced rats. Additionally, both doses of extracts improved (*p* < 0.05) the DI values of LPS-treated rats. There were no substantial differences between the groups of rats in the exploration time of a familiar object (FO1) during the test session. 

### 2.5. MASE Improved the Cholinergic Functions of LPS-Induced Rats

**Figure 3:**
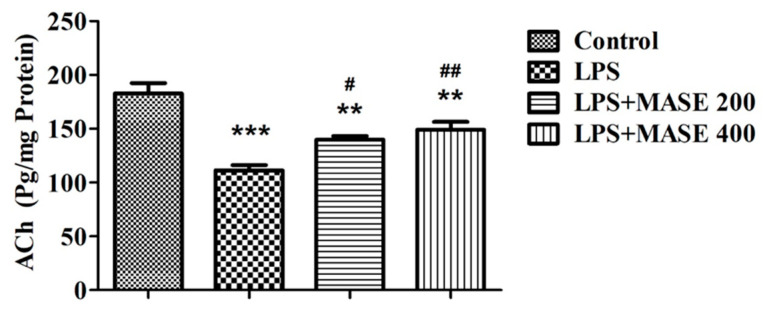
Effect of methanolic Ajwa seed extract (MASE) on brain acetylcholine (ACh) levels of LPS-induced rats. The results are expressed by mean ± SEM (*n* = 6). One-way ANOVA [F(3,20) = 19.59, *p* < 0.001] followed by the Tukey–Kramer multiple comparisons test for comparisons within the groups. ** *p* < 0.01 and *** *p* < 0.001 as compared to the control group; ^#^ *p* < 0.05 and ^##^ *p* < 0.01 as compared to LPS-induced group.

Acetylcholine (ACh) levels were measured in the brain homogenate of treated rats (Figure 3). Following a one-way ANOVA, a variation in ACh levels [F(3,20) = 19.59, *p* < 0.001] was observed between the different treated groups. A post-hoc examination of the selected groups revealed that LPS-induced rats had extensively (*p* < 0.001) lower ACh levels than the control group. However, compared to the group that was just LPS-induced, thirty days of MASE administration with both doses considerably improved the cholinergic activity by increasing the level of ACh (*p* < 0.05 with 200 mg/kg, and *p* < 0.01 with 400 mg/kg, p.o.). The reversal of ACh levels with MASE was not comparable with the control group. There were significant deviations (*p* < 0.01) in the ACh levels associated with the control animals. 

### 2.6. MASE Ameliorates the Neuroinflammatory Parameters of LPS-Induced Rats

**Figure 4:**
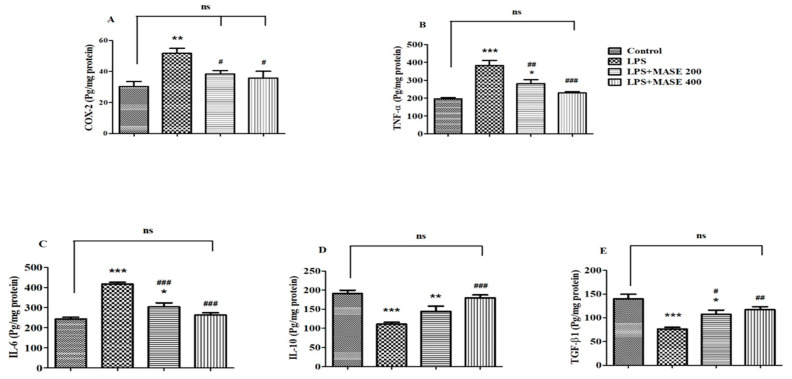
Effect of methanolic Ajwa seed extract (MASE) on (**A**) COX-2, (**B**) TNF-α, (**C**) IL-6, (**D**) IL-10, and (**E**) TGF-β1 of LPS-induced rats. The results are expressed by mean ± SEM (*n* = 6). One-way ANOVA [F(3,20) = 7.414, *p* < 0.01 for COX-2; F(3,20) = 19.77, *p* < 0.001 for TNF-α; F(3,20) = 36.40, *p* < 0.001 for IL-6; F(3,20) = 15.25, *p* < 0.001 for IL-10; and F(3,20) = 11.85, *p* < 0.001 for TGF-β1] followed by the Tukey–Kramer multiple comparisons test for comparisons within the groups. * *p* < 0.05, ** *p* < 0.01, and *** *p* < 0.001 as compared to the control group; ns—not significant as compared to the control group; ^#^ *p* < 0.05, ^##^ *p* < 0.01, and ^###^ *p* < 0.001 as compared to the LPS-induced group.

The levels of the cyclooxygenase-2 (COX-2) enzyme and targeted cytokines listed as TNF-α, IL- 6, IL-10, and TGF-β1 were evaluated using the brain tissues from the treated rats (Figure 4). A one-way ANOVA revealed a significant effect of MASE on COX-2 levels [F(3,20) = 7.414, *p* < 0.01] in LPS-induced rats (Figure 4A). The injection of LPS caused an elevation of COX-2 levels (*p* < 0.01) when compared to the control group. Nevertheless, MASE treatment decreased (*p* < 0.05) the COX-2 activity at both dose levels (200 and 400 mg/kg, p.o.) in comparison with LPS-induced rats. 

A significant change in both pro-inflammatory cytokine levels [TNF-α; F(3,20) = 19.77, *p* < 0.001 and IL-6; F(3,20) = 36.40, *p* < 0.001] was seen when matched between all the groups. The LPS-induced group revealed an elevation (*p* < 0.001) in TNF-α levels compared to the control group. Oral administration of MASE, however, considerably decreased the brain’s TNF-α levels compared to the LPS-induced group (*p* < 0.01 and *p* < 0.001 for 200 and 400 mg/kg, respectively) (Figure 4B). Similarly, LPS-induced rats exhibited considerable elevation (*p* < 0.001) of IL-6 levels in their brains compared to the control rats, while the administration of MASE considerably restored (*p* < 0.001) the elevated IL-6 levels at both dose levels compared to the LPS-induced group (Figure 4C). The reduction of both pro-inflammatory cytokine levels was greater in a high dose (400 mg/kg, p.o.) of MASE-treated animals.

Referring to Figure 4D,E, a one-way ANOVA analysis highlighted the significant changes in both targeted anti-inflammatory cytokine levels [IL-10; F(3,20) = 15.25, *p* < 0.001 and F(3,20) = 11.85, *p* < 0.001 for TGF-β1] among all the groups. Additional extended analysis between the two selected groups explained a major decline (*p* < 0.001) in both anti-inflammatory cytokine levels, such as IL-10 and TGF-β1, as related to the control group. Compared to the LPS-induced group, the IL-10 levels in the brain dramatically improved with a higher dose of MASE (400 mg/kg, p.o.). Regarding brain TGF-β1 levels, both doses of MASE restored the TGF-β1 levels (*p* < 0.05 with 200 mg/kg and *p* < 0.01 with 400 mg/kg, p.o.) in LPS-treated rats.

### 2.7. Molecular Docking

Our results indicated improvement in the ACh levels and anti-inflammatory parameters with a decrement in proinflammatory parameters. The phytochemical analysis indicated the presence of a significant amount of phenolic compounds with a considerable amount of flavonoids and tannins. The literature search showed ellagic acid, gallic acid, pyrogallol, benzoic acids, protocatechuic acid, cinnamic acid, catechol, vanillic acid, syringic acid, catechin, and epicatechin are the phenolics present in methanolic extracts of seed from *Phoenix dactylifera* L. The flavonoids reported in the methanolic extract are hesperidin, narengin, rutin, hesperetin, quercetin, kaempferol, and apigenin [26]. Therefore, the above-mentioned compounds are considered for molecular docking studies. Our previous studies using an aqueous extract of Sukkri dates and a target fishing approach together indicated acetylcholinesterase (AChE) and COX as potential targets for our observed results [20]. Therefore, molecular docking was performed for the above-mentioned compounds using AChE (PDB code: 1DX6) and COX-2 (PDB code: 5IKR). The docking methodology was validated by reproducing the binding mode of co-crystallized ligands and was performed following previous reports. The cocrystallized ligands displayed a binding affinity of −10.5 and −9.1 kcal/mol for AChE and COX-2, respectively. Table 2 displays the binding affinity for the docked ligands using the crystal structures of AChE and COX-2. The phytochemicals present in MASE displayed binding affinity in the range of ~−11.0 kcal/mol to ~5 kcal/mol for AChE.

It should be noted that all the flavonoids, including hesperidin, hesperetin, kaempferol narengin, quercetin, rutin, and apigenin, displayed binding affinity comparable to cocrystallized ligands. Similarly, among the phenolic compounds, ellagic acid and epicatechin displayed binding affinity comparable to co-crystallized ligands. The calculated binding affinity for COX-2 was found in the range of −9.0 to ~−3.0 kcal/ mol. among the flavonoids (kaempferol, quercetin, and apigenin), which displayed binding affinity comparable to the co-crystallized ligand. Among the phenolics, epicatechin and catechin displayed significant binding affinity. 

**Figure 5:**
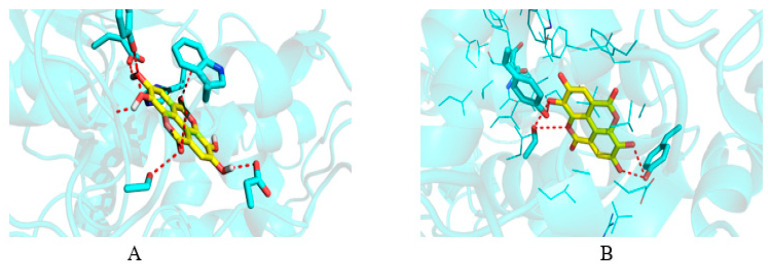
Docked pose of ellagic acid into the cavity of (**A**) AChE and (**B**) COX-2.

In order to identify molecular-level interactions, binding mode analysis was performed for those compounds that displayed binding affinity of −7 or above for AChE and −6 or above for COX-2; those binding modes were selected for discussion, which displayed interaction with key residues. The AChE literature search and crystal structure analysis revealed Tyr70, Asp72, Trp84, Gly118, Gly119, Tyr121, Ser200, Ala201, Trp279, Glu337, and His440 [27,28,29]. Similarly, for COX-2, the literature search and crystal structure analysis revealed Arg120, Ser530, Tyr355, Tyr385, Ala527, Val349, Leu352, Leu531, and Trp387 [30]. Table 3 and Table 4 display the detailed residue-wise interaction for the docked ligands in the complexes of AChE and COX-2. Figure 5 shows the docked pose of ellagic acid into the cavity of AChE and COX-2. 

### 2.8. Molecular Dynamics

**Figure 6:**
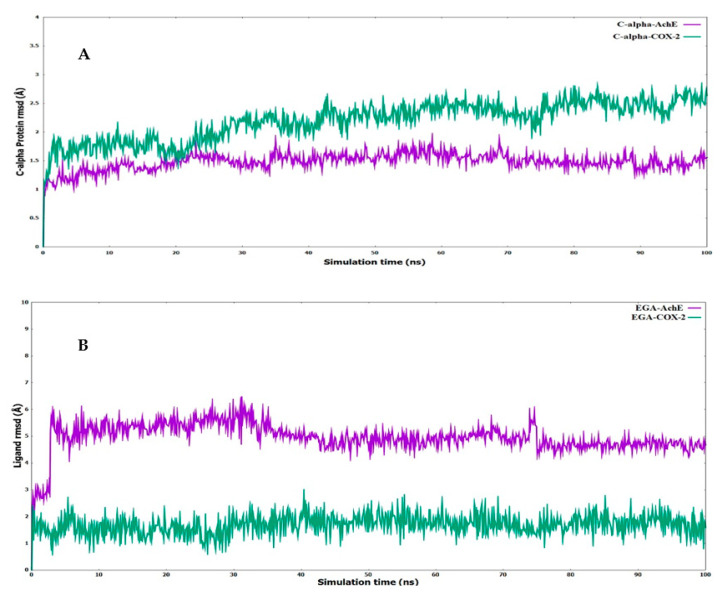
Root mean square deviations (RMSD) for the (**A**) α-carbon chain of AChE and COX-2 (**B**) ligand (EGA—ellagic acid) complexed with AChE and COX-2.

**Figure 7:**
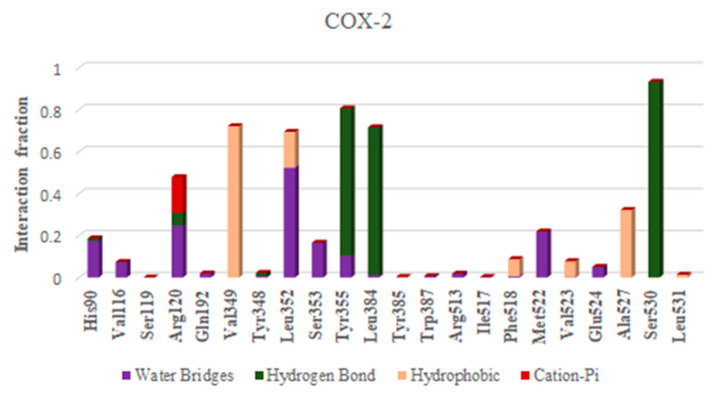

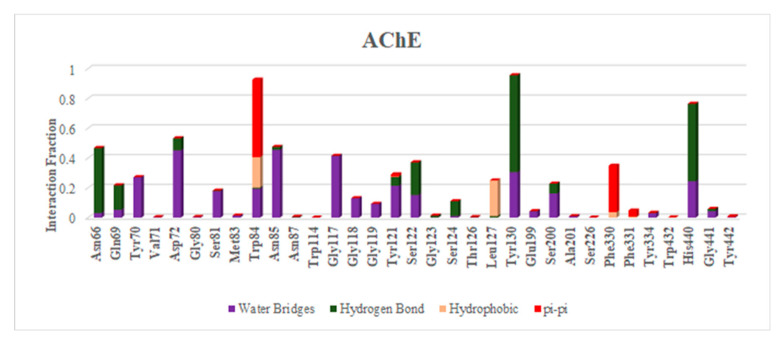
Histogram of interaction COX-2 and AChE with ellagic acid during MD of 100 ns.

Depending upon the binding mode analysis and binding affinity, the best pose of ellagic acid was obtained from molecular docking and used for generating protein–ligand complexes. The molecular dynamics simulations for these protein–ligand complexes (COX-2-Ellagic acid and AChE-Ellagic acid) were carried out for 100 ns at 300 K using Desmond software. In MD studies, the stability of protein and ligand in complexes is determined by computing root mean square deviations (RMSD). Figure 6A shows the RMSD of both COX-2 and AChE’s Cα atoms of backbone, and Figure 6B depicts the RMSD of ellagic acid in the active sites of both proteins for a duration of 100 ns. The average RMSDs of Cα atoms are 2.20 Å and 1.48 Å, respectively, for COX-2 and AChE, indicating the enzymes are stable during simulation. The RMSDs of ellagic acid for the complexes of COX-2 and AChE are 1.69 Å and 4.96 Å, respectively. The higher RMSD value for ellagic acid in the AChE enzyme is due to its movement from docking binding pose to a new binding mode during MD simulation within the first 10 ns of simulation. However, the new binding mode did not display any significant changes for a further period of simulations. Figure 7 shows detailed 2D interactions of ellagic acid with COX-2 and AChE, including hydrogen bonding, hydrophobic, etc., changed during the simulations. These reported interactions in the figure appeared in more than 30% of the simulation time. In the active site of COX-2, ellagic acid forms direct hydrogen bonding interaction with residues Tyr355, Tyr385, and Ser530 and makes an indirect hydrogen bond with Leu352. One of the hydroxyl groups of ellagic acid forms a direct hydrogen bond with Tyr355, which appears for 70% of the simulation time. Another hydroxyl group makes two hydrogen bond interactions with the Ser530 and Tyr385 residues. It is to be noted that this hydroxyl group acts as a hydrogen bond donor and acceptor with the Ser530 and Tyr385 residues, respectively. The interaction with Ser530 appears for 78% simulation time, during interaction with Tyr385 for 43% time of the simulation run. Leu352 is involved in a water-mediated hydrogen bonding interaction with one of the carbonyl groups of ellagic acid for 49% of the simulation time. In the complex of ellagic acid with AChE, a hydroxyl group of ellagic acid had an interaction with Tyr130 (hydroxyl group) up to 2.7 ns. After that, the oxygen atom of the carbonyl group of ellagic acid forms direct hydrogen bonding interaction with the hydroxyl group of aromatic residues Tyr130, which stays for 62% of simulation time, up to 70 ns. In the last 30 ns, ellagic acid breaks this hydrogen bonding interaction and makes a π–π interaction with the Tyr and Trp residues in such a way that ellagic acid is sandwiched between the Phe330 and Trp84 residues. Additionally, two hydroxyl groups of ellagic acid show hydrogen bonding interactions with the backbone carbonyl group of His440. The hydroxyl group from another end of ellagic acid also forms a hydrogen bonding interaction with ASP 72, but less frequently than His440 during the last 30 ns. Ellagic acid complexed with COX-2 had binding free energies of −55.84 kcal/mol and −58.09 kcal/mol when the unbound states of the enzyme and ligand were relaxed and not relaxed, respectively. 

**Figure 8:**
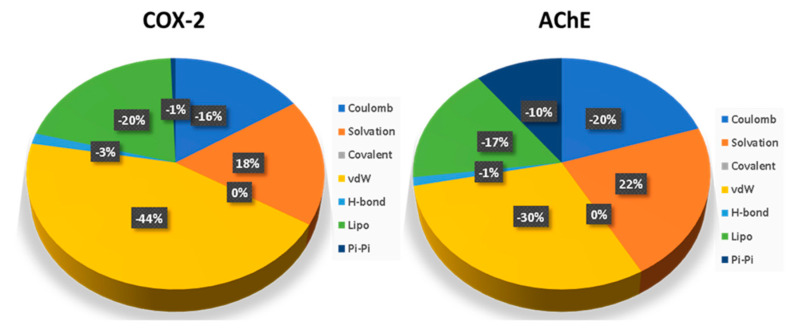
Contribution of basic interactions to binding free energies of bound ellagic acid in COX-2 and AChE.

The major contribution to the energy for ellagic acid binding to COX-2 comes from vdW, lipophilic and Coulombic interactions (Figure 8), where vdW contributes around 44%, followed by lipophilic interaction (20%). Ellagic acid’s binding free energies with AChE were −70.69 kcal/mol and −72.07 kcal/mol, respectively, in the relaxed and unrelaxed unbound states of the enzyme and ligand. In the case of the ellagic acid bound to AChE, significant contributions are from vdW, lipophilic and Coulombic interactions, where VdW contributes significantly, followed by the Coulombic interaction. The solvation penalty associated with AChE is around 4% higher than COX-2.

## 3. Discussion

Cytokines and interleukins exacerbate the processes that activate inflammatory pathways in neurodegenerative disorders, such as AD, multiple sclerosis, Parkinson’s disease, and amyotrophic lateral sclerosis. LPS is a neurotoxin and is widely used as an experimental model for inducing the systemic inflammatory process. This study examined the effect of MASE on LPS-induced deficiency in learning ability and memory capability, cholinergic deficiency, and pro-inflammatory as well as anti-inflammatory cytokine alterations in experimental rats. Our results showed an improvement in spatial learning and memory by administration of MASE using selected maze models, indicating that it alleviated cognitive deficits induced by neuroinflammation in LPS-treated rats. Protection of cognitive functions was associated with improvement of cholinergic neuronal functions, decreased COX-2 enzyme activities as well as pro-inflammatory cytokines, and improved anti-inflammatory cytokines in the LPS-induced rat brain.

According to our previous findings, four doses of peripheral injections of LPS (250 µg/kg, i.p.) caused memory dysfunctions in rodents by generating neuroinflammation in the brain, thereby modifying COX activity and cytokine levels [6,31]. In the present study, the induction of neuroinflammation in the rat brain was shown by the increase of COX-2, TNF-α, and IL-6 production and reduction of TGF-β1 and IL-10 levels. Parallel to these results, the neuroinflammation status of multiple LPS intraperitoneal injections was associated with the elevation of pro-inflammatory cytokines, including IL-6 and TNF-α, as well as a decline of anti-inflammatory cytokine IL-10 in the rat’s hippocampus [32]. 

Furthermore, LPS administration increased the release of inflammatory mediators such as NF-κB, TNFα, Toll-like receptor 4 (TLR4), and IL-6 in the rats’ brains and also reduced brain-derived neurotrophic factor (BDNF) [33]. Additionally, the current results showed that LPS declined cognitive parameters such as lengthening the TL performance in the EPM test, reducing the exploration time of a novel objective (NO) and the percentage of discrimination index (DI) in the NOR test, and deteriorating the brain’s ACh levels. Numerous studies have established the connection between the inflammatory process induced by LPS and cognitive changes. Using passive avoidance (PA) and Morris water maze (MWM) tests, LPS administration resulted in the impairment of memory functions of rats by altering the targeted parameters [32]. Subsequent intraperitoneal LPS administration also confirmed the impaired spatial recognition memory in various behavioral models, including the Y-Maze, NOR test, PA tasks, and EPM test [6,31,34]. 

In this study, the effectiveness of MASE in improving memory deficits caused by LPS treatment in rats was assessed using various maze models such as the EPM and NOR tests. Among other things, the EPM assay evaluates TL by measuring cognitive and memory functions such as long-term spatial memory. During EPM exploration, the animals avoid staying in open as well as elevated regions, preferring closed and dark areas. These reflect the shorter TL values supporting the improvement of spatial memory [3,31]. As previously stated, a longer TL of both acquisition and retention sessions on day 1 and day 2 in LPS-induced rats linked to the control group described the impairment of memory with LPS treatment. The administration of MASE (200 and 400 mg/kg, p.o.) decreased the TL duration of retention (day 2) in a dose-dependent manner in the LPS-induced rat model. 

According to a NOR test, the same doses of MASE treatment ameliorated the LPS-induced exploration time of a NO and the percentage of DI during the test session, when the animals were allowed to explore both a familiar object (FO1) and an NO. To discriminate between the two objects, the animals first attended to two identical objects (FO1 and FO2) in an early test session for working memory [31]. In the test session, the animals spent longer with the NO than the FO, indicating the discrimination ability of an NO, as well as recalling the FO from the prior test session. The NOR task requires the use of additional cognitive skills, such as studying a single unfamiliar object or a task in a novel location. When the animals are allowed to approach an NO and an FO, they approach the novel object more frequently and prefer to spend more time examining it than the familiar one, as previously documented [35,36]. Furthermore, the treatment groups’ higher DI provides further proof of the animals’ discrimination capacity during the test session. 

There is currently a lack of evidence about the effects of dates on cognitive performance. Comparable to our results, thirty-day oral administration of aqueous Ajwa seed extract (200 and 400 mg/kg) to type-2 diabetes mellitus rats resulted in improvement of various cognitive parameters such as shortened TL values in the EPM, prolonged exploration time of targeted NO, and improved DI between two objects (FO and NO) in the NOR test. Additionally, in the Y-Maze test, an enhancement in the number of novel arm entries, coping behavior, and curiosity behavior in type-2 diabetes mellitus rats was seen [18]. In addition, continuous four-month feeding of 2% and 4% date fruit pellets to APP-transgenic Alzheimer’s mice resulted in considerable memory improvement, decreased anxiety-like behavior, reversal of spatial learning capacity, position discrimination learning capability, and motor coordination. Both Aβ1–40 and Aβ1–42 levels were also drastically lowered with a supplement of date fruits [17]. Date seed extract from Bam Mazafati Rutab restored learning and memory of β-amyloid-induced impairments in rats, according to the MWM test [37].

ACh, a major neurotransmitter in the neurological system, has long been related to various memory functions. A deficiency in the hippocampus’ ACh levels has been known to be related to age-related cognitive decline, and the release of Ach in the hippocampus is increased when carrying out spatial memory tasks [38]. Moreover, the cholinergic system has been shown to play a significant role in the regulation of internal immunological response as well as neuro-immune interactions. Numerous animal studies have established that LPS induction results in the impairment of memory performance via the reduction of ACh and elevating the activity of its metabolic enzyme AChE [31,39]. Our previous findings sustained the present results, as thirty days of treatment with aqueous Ajwa seed extract improved ACh levels in diabetic-induced rats’ brains by decreasing AChE activity [18]. In LPS-stimulated human macrophage cells, ACh significantly inhibited the release of cytokines such as IL-1β, TNF-α, and IL-6 [40]. In the present study, the elevation of ACh levels by treatment with MASE might also be due to the reduction of cytokine levels (TNF-α and IL-6) in rats pretreated with the extract followed by LPS induction.

Evidence suggests that in AD, inflammatory damage of neurons and glial cells induces the formation as well as aggregation of Aβ protein and that addressing neuroinflammation is a crucial factor in the slow progression of neuronal degradation in AD. Moreover, neuroinflammation creates an abnormal release of proinflammatory cytokines, which stimulates signaling pathways that promote brain tau hyperphosphorylation in residues that are not altered in normal physiological circumstances [41]. In clinical and pre-clinical models of dementia and AD, neuroinflammatory mediators such as the COX enzyme and pro-inflammatory cytokines, including TNF-α, IL-6, IL-1α, and IL-1β, trigger the nervous system’s inflammatory responses [42]. COX-2, one of two COX isoenzymes, is a well-known inducible enzyme that responds to inflammatory stimuli such as cytokines and mitogens. The activation of cytokines, such as TNF-α, IL-1β, and IL-6, has been shown to increase COX-2 expression in microglia [43]. 

In a recent study, an in vitro exposure of LPS in BV2 cells and primary microglia cells resulted in the liberation of pro-inflammatory factors, like COX-2, iNOS, NO, PGE2, TNF-α, IL-6, and IL-1β [44]. Moreover, single or multiple doses of LPS injection can produce a cascade of cellular destruction due to the deregulation of immune-inflammatory responses to infective stimuli. In the cerebral cortex and hippocampus of presenilin PS1 mutant mice, LPS induced considerably higher levels of TNF-α, IL-1α, IL-1β, IL-6, and mRNAs expression as compared to wild-type animals [45]. Moreover, the level of NF-κB expression in the prefrontal cortex and hippocampus of rats’ brains was likewise significantly increased after LPS administration [46]. A previous report from our laboratory also showed an elevation of COX-2, IL-6, and TNF-α levels after LPS induction in mice brains [6]. Similar to earlier reports, the present four systemic injections of LPS considerably increased COX-2 enzyme activity and targeted pro-inflammatory cytokines such as TNF-α and IL-6 levels in the rat brain. However, thirty days of oral administration of MASE (200 and 400 mg/kg) ameliorates LPS-induced COX-2, TNF-α, and IL-6 levels successfully in animals from test groups. Previously, treatment with aqueous extract from Ajwa seed was seen to significantly decline brain COX-2, IL-6, and TNF-α levels in type 2 diabetic-induced rats [19].

On the other hand, there is ample evidence of anti-inflammatory cytokine action on neuroprotection in animal models. Four consecutive injections of LPS (250 µg/kg, i.p.) in mice suppressed the action of anti-inflammatory cytokine markers like TGF-β1 and IL-10 in the brain, according to a recent study [6]. Furthermore, several other investigations have found that LPS induction reduces the levels of both cytokines IL-10 and TGF-β1 [47,48]. Interestingly, each anti-inflammatory cytokine contributes to neuroprotection in a different way. Limitation of excessive inflammatory responses, activation of innate immunity, and encouragement of tissue healing mechanisms are all linked to the anti-inflammatory responses of cytokine IL-10. It can also control the production of pro-inflammatory cytokines like IL-1β and TNF-α, as well as reduce cytokine receptor expression and inhibit receptor activation in the brain [49]. TGF-β1 is another important cytokine that regulates cell differentiation, proliferation, and the production of extracellular matrices. It also prevents T and B cells from proliferating and differentiating, as well as the production of IL-2, IL-17, TNF-α, and IFN- γ [50]. In a previous study with type-2 diabetic rats, treatment with an aqueous extract of Ajwa seed was shown to increase brain TGF-β1 and IL-10 levels [19]. Our current findings showed that LPS administration was connected to lower levels of anti-inflammatory cytokines, like TGF-β1 and IL-10, in the rat brain, while a larger dose of MASE (400 mg/kg, p.o.) was linked to higher levels of both of the above cytokines in the rat brain. Treatment improved both cytokine levels in the brain, indicating that it has anti-inflammatory properties.

Phytochemical analysis revealed the presence of a significant amount of phenolics and a considerable amount of flavonoids and tannins. A literature search showed ellagic acid, epicatechin, catechin, pyrogallol, syringic acid, vanillic acid, benzoic acid, catechol, cinnamic acid, and gallic acid as the phenolic compounds, while hesperidin, hesperetin, kaempferol, narengin, quercetin, rutin, and apigenin are the flavonoids commonly found the methanolic extract of dates seed [26]. The experimental results in this study showed that administration of two doses of MASE resulted in improved levels of ACh, IL-10, and TGFβ1 while causing a decrement in the levels of COX-2, TNF-α, and IL-6 at the same time. Our previous reports and target fishing using PharmMapper [51] suggested AChE and COX as the potential targets for our observed results; hence, the above-mentioned molecules were docked into the active site using AChE and COX. The results of molecular docking were evaluated based on the calculated binding affinity and interactions with key residues. The key residues considered for AChE were Tyr70, Asp72, Trp84, Gly118, Gly119, Tyr121, Ser200, Ala201, Trp279, Glu337, and His440. For COX-2, Arg120, Ser530, Tyr355, Tyr385, Ala527, Val349, Leu352, Leu531 and Trp387 were considered important for binding mode analysis. In the case of AChE residues, Ser200, Glu337, and His440 form the catalytic triad, while residues Gly118, Gly119, and Alanine together make the oxyanion binding sites [27,28]. The peripheral aromatic binding site is made up of Asp72. Trp84, and Trp279 [29]. Depending upon the binding affinity displayed for AChE (−7 or above), ellagic acid, epicatechin, catechin, cinnamic acid, hesperidin, hesperetin, kaempferol, nerengin, quercetin, rutin, and apigenin was selected for binding mode analysis. It is to be noted that the above-mentioned ligands, except rutin, displayed hydrogen or hydrophobic interactions with residues Asp72, Trp84, Glys118, Ser200, Tyr334, and His440. Furthermore, the Asp72, Gly118, Ser200, and His440 residues are important components in the catalytic machinery responsible for the hydrolysis of ACh, while Trp84 and Tyr334 residues play important roles in the binding of substrate and ligands. It is pertinent to mention that rutin, despite showing a significant binding score, did not display interactions with the key residues considered for the study. In the case of COX-2, ellagic acid, epicatechin, catechin, kaempferol, quercetin, and apigenin were considered for binding mode analysis as their binding affinity was above −7 kcal/mol. The binding mode analysis reveals interactions with residues Val349, Leu352, Tyr385, Tyr355, Ala527, Ser530, and Leu531. It is pertinent to mention that the above-mentioned residues of COX-2 are part of a hydrophobic channel through which arachidonic acid passes and reaches the oxygenation site of COX [30].

In order to test the stability of ligands under dynamical conditions, a complex of ellagic acid with AChE and COX-2 was chosen for molecular dynamics of hundred nanoseconds. The simulation run of the AChE complex showed changes in the binding mode of ligand during the initial phase of the simulation run; however, the ellagic acid displayed significant stability in the last 30 s of the simulation run and displayed hydrogen bond interaction with Asp72 and His440 for a considerable period of simulation time. The COX-2 complexed with ellagic acid displayed significant stability of ligands in the hydrophobic channel and maintained the hydrogen bond interaction with Leu 352, Tyr355, Tyr385, and Ser530 during a significant period of the simulation run. Therefore, considering the results for molecular modeling, it can be stated that improvement in the cholinergic transmission is due to the inhibition of AChE by the compounds ellagic acid, epicatechin, catechin, cinnamic acid, hesperidin, hesperetin, kaempferol, quercetin, rutin, and apigenin, while improvement in the inflammatory conditions is due to the blockage the hydrophobic channel of COX-2 by the compounds ellagic acid, epicatechin, kaempferol, quercetin, and apigenin. Our results from the molecular dynamics is in line with the literature reviewed by Banc et al. for ellagic acid. The report states that plant sources rich in ellagic acid and its derivatives display their neuroprotective effect through the inhibition of a COX-mediated inflammatory pathway [52].

## 4. Materials and Methods

### 4.1. Plant Extraction

Ajwa dates were obtained from the local dates market of Al-Madinah, Saudi Arabia. The obtained dates were identified using polymerase chain reaction (PCR) by Professor Mohamed Motawei, College of Agriculture and Veterinary Medicine, Qassim University. The seeds were removed from the fruit pulp and thoroughly rinsed in water to remove all the pulp. The collected seeds were left to dry for 2–3 days at room temperature. Finally, the coarse powder was prepared from the dried hard seeds using a coffee seed grinder. Each 100 g of coarse powder was soaked in one liter of methanol (99.8%; SRL Chemicals, Maharashtra, India) at room temperature for three days. A rotary evaporator was used to condense the liquid extract at 40 °C. The collected methanolic Ajwa seed extract (MASE) was freeze-dried and preserved for future research.

### 4.2. Quantitative Assessment of the Total Polyphenolics, Total Flavonoids, and Total Tannins

The quantitative spectrophotometric assays were used to measure the total phenolic contents (TPC), total flavonoid contents (TFC), and the total tannins contents (TTC) in MASE using standard calibration curves of the gallic acid, quercetin, and catechin, and calculated equivalents to gallic acid (GAE), quercetin (QE), and catechin (CE), respectively.

#### 4.2.1. Total Phenolic Contents (TPC)

In this method, the diluted Folin–Ciocalteu reagent (1:5 in distilled water, 0.2 mL) was mixed with 0.2 mL of 10% *w*/*v* sodium carbonate and 1.6 mL of the MASE (final concentration equal to 0.1 mg/mL). The mixture was incubated for 30 min at room temperature. The developed blue color was measured at 760 nm. Total polyphenolic levels were measured in triplicates and represented as gallic acid equivalent (GAE) per gram of dried MASE [23].

#### 4.2.2. Total Flavonoid Content (TFC)

The contents of MASE flavonoids were assessed according to the literature methods [23,51]. A 100 µL potassium acetate (0.1 mM) and 100 µL of 10% solution of aluminum chloride in distilled water were added to 2000 µL of MASE (0.1 mg/mL) in test tubes. The mixtures were thoroughly mixed and kept in a dark place at room temperature for 30 min. The absorbance of the resulting color was measured at 415 nm. Three independent measurements were used to compute the total flavonoid contents, which were then represented as quercetin equivalent per gram of the dried MASE.

#### 4.2.3. Total Tannins Contents (TTC)

The MASE total tannins content was measured by the method described in the literature by Mani et al. (2022) [20]. A mixture of 1.5 mL of the vanillin solution (4% in methanol) and 750 µL of the concentrated HCl was added to Ajwa extract (200 µL of the 0.1 mg/mL) in a test tube. The tube content was thoroughly mixed and kept in the dark place at room temperature for 15 min. At 500 nm, the absorbance was measured. The total tannin contents were assessed from three independent measurements and represented as catechin equivalent per gram of the dried ASSE.

### 4.3. Experimental Animals

A total of twenty-four male Sprague Dawley (SD) rats (3 months old; 150–200 g body weight) were obtained from the College of Pharmacy’s animal facility at Qassim University for this study. In the acute toxicity study, a similar age match of female SD rats was employed. The (U.S.) National Research Council’s Guide for the Care and Use of Laboratory Animals was followed for all animal research. Qassim University’s Deanship of Scientific Research’s Committee on Health Research Ethics granted bioethical authorization (protocol number 21-10-02) for the current experimental procedures. The animals were maintained in standard laboratory conditions (25 ± 1 °C) and relative humidity (60 ± 5%) with a 12 h day–night cycle for acclimatization (7 days) and during the experimental procedure (30 days). Rats were allocated into four groups at random, with each group consisting of six individuals. The rats were housed three in a polypropylene cage. They were given free access to water and standard pellet rodent food from First Milling Company, Jeddah, Saudi Arabia, during the entire experiment.

### 4.4. Vehicle

MASE was prepared as a suspension in 0.5% (*w*/*v*) carboxymethyl cellulose sodium (CMC) and administered to the animals orally (p.o.). A neurotoxin, lipopolysaccharide (LPS; Sigma-Aldrich Co, St. Louis, MO, USA) was diluted in 0.9% (*w*/*v*) normal saline and given intraperitoneally (i.p.).

### 4.5. Acute Toxicity Study

The Organization for Economic Co-operation and Development (OECD-423) guidelines for acute oral toxicity—acute toxic class method—was followed for the acute toxicity studies [53]. Using a random sample procedure, three female rats were assigned to each step in this experiment. The animals had fasted overnight, and MASE was given orally at a dose of 5 mg/kg at first. For the first four hours, toxic symptoms were carefully observed, and mortality was monitored for an additional three days. The dose was considered toxic when a minimum of two out of three animals died and, if one of three animals died, the same dose was given again to confirm the toxic impact. There was no mortality at the lower dose; thus, higher doses of MASE (50, 300, and 2000 mg/kg, p.o.) were used to confirm the toxicity, according to the guidelines [18].

### 4.6. Experimental Design

**Figure 9:**
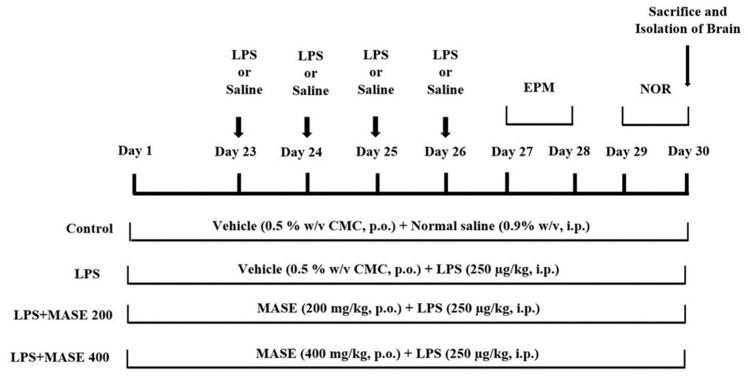
Timeline administration of the drugs, behavioral assessments, and isolation of brain samples. The groups of rats were administered vehicle or methanolic Ajwa seed extract (MASE) for thirty days orally. Except for the control, other groups were injected with four doses of LPS (250 μg/kg, i.p.) for inducing neuroinflammation (days 23–26). Regarding elevated plus-maze (EPM) assessments, the training sessions were conducted on day 27, and memory assessments were analyzed on day 28, whereas both sessions of the novel object recognition (NOR) test were conducted on day 29 and day 30, respectively. At the end of the memory tests, on day 30, all the animals were sacrificed and brain tissues were collected for ELISA tests.

The total number of twenty-four male rats was divided into four groups, so each group consisted of six rats. In the control group (I), rats were treated orally with the vehicle (0.5% *w*/*v* CMC) for 30 days and were given four injections of normal saline (0.9% *w*/*v*, i.p.) from day 23 to day 26. In the LPS-induced group (II), the rats were treated orally with the vehicle (0.5% *w*/*v* CMC) for 30 days and were given four injections of LPS (250 µg/kg, i.p.) from day 23 to day 26. In the test groups (III and IV), the rats were treated orally with MASE 200 mg/kg or 400 mg/kg, respectively, for 30 days, and were given four injections of LPS (250 µg/kg, i.p.) from day 23 to day 26. The doses of MASE (200 and 400 mg/kg, p.o.) were chosen based on the findings of our acute toxicity experiments and in parallel with our prior work with aqueous Ajwa seed extract [18]. The dose of LPS (250 µg/kg, i.p) was also determined from a research report from our laboratory [6]. For the spatial memory assessments of rats, an elevated plus-maze test was performed on days 27 and 28, and a novel object recognition (NOR) test was performed on days 29 and 30 of the drug treatment schedule. At the end of the drug treatment and tests (day 30), all the animals were sacrificed for the preparation of the brain homogenate (Figure 9).

### 4.7. Elevated Plus Maze (EPM) Test

The EPM assay analyzes cognitive and memory functions, such as long-term spatial memory, to assess transfer latency (TL) [3,18]. The TL is the time it takes a rat to move from an open arm to either one of the closed arms using all four feet (in seconds). The EPM was made of wood and stood 50 cm above the ground. It consisted of two open and two closed arms. All four arms were equal in size (50 × 10 cm^2^) and the two closed arms were surrounded by 40 cm high walls. The test was conducted in two sessions: training (day 27) and retention (day 28). During the training session, each rat was positioned at the end of the open arm, away from the central platform, and the TL was recorded. If the rat did not enter either of the closed arms after 90 s, it was gently guided into one and given another two minutes to explore the maze. After 24 h, the TL was measured as the retention of learned-task memory (day 28).

### 4.8. Novel Object Recognition (NOR) Test

By altering the technique, the NOR task can be used to explore diverse rodent behaviors such as short-term, intermediate-term, and long-term memories [18,54]. The present test was performed using an open wooden box apparatus (80 × 60 × 40 cm). Two similar familiar objects (FO1 and FO2; cylindrical box) and one novel object (NO; rectangle box) were used for testing the discrimination of the objects. The procedures were divided into three stages: habituation, training, and testing (days 29 and 30 of drug treatment). During the habituation procedure, each animal was given 5 min to explore the box without any objects (day 29). The training session was conducted after 24 h (day 30) of habituation, and each rat was given five minutes to explore two similar cylindrical boxes (FO1 and FO2). After a four-hour break, the test session was scheduled. In this test session, each animal was again allowed to explore with another set of two objects (FO and NO) for 5 min. During the test session, the time spent exploring the FO, as well as the NO, was recorded. An animal’s exploration time was defined as the amount of time it spent pointing its nose at an object at a distance of less than or equal to 2 cm. The discrimination index (DI) was calculated as follows: DI = N − F/N + E, where N refers to the exploration time of the NO, and F—refers to the exploration time of the FO [18].

### 4.9. Collection of Brain Samples

All of the rats were sacrificed by cervical decapitation under light ether anesthesia on day 30 of the NOR test, and the entire brain was carefully separated from the skull. The separated brain tissues were minced in ice-cold phosphate buffer (pH 7.6). Then the collected homogenate was centrifuged at 4000 rpm for 10 min, and the cloudy supernatant fluid was utilized to assess the cholinergic and neuroinflammation parameters as described below. To evaluate the total protein content of the samples, biuret colorimetric analysis was utilized (Crescent Diagnostics, Jeddah, Saudi Arabia).

### 4.10. Enzyme-Linked Immunosorbent Assay (ELISA) of Cholinergic and Inflammatory Parameters

Using specific ELISA kits, the levels of ACh, TNF-α, IL-6, IL-10, TGF-β1, and COX-2 were estimated as described in the manufacturer’s protocol (Cloud-Clone Corp., Texas, USA). Briefly, after preparing all reagents, samples, and standards, 100 μL of standard or sample was added to the targeted antibody-coated well and incubated for 1 h at 37 °C. After aspirating the plate, 100 μL of prepared detection reagent A was added. After incubation for 1 h at 37 °C, the plate was washed three times. Next, 100 μL of detection reagent B was added, incubated at 37 °C for 30 min, and then washed five times. Finally, 90 μL of substrate solution followed by 50 μL of stop solution was added to each well. The intensity of color development was immediately read at 450 nm (BioTek microplate reader, BioTek Instruments, Winooski, VT, USA).

### 4.11. Statistical Analysis

The results were specified as mean ± standard error mean (SEM). The significant levels between the groups were calculated using a one-way ANOVA analysis and the Tukey–Kramer post hoc test (Graph Pad version 9.0, GraphPad Software Inc., San Diego, CA, USA). Statistical significance was defined as a *p*-value of less than 0.05.

### 4.12. Molecular Modelling

Crystal structures with PDB code 1DX6 and 5IKR for AChE and COX-2, respectively, were downloaded from the Protein Data Bank. The required input files were prepared using AutoDock tools bundled with MGL tools (version 1.5.6, La Jolla, CA, USA). The proteins were prepared by removing water molecules, heteroatoms, and any extra chain if present. Missing residues were added, followed by the addition of polar hydrogens and Kollman charges. The protein molecules were then converted into pdbqt format. Three-dimensional structures of ligands were downloaded from the PubChem database in SDF format. The downloaded structures were minimized using the MM94 force field, Gasteiger charges were added, and torsions were defined followed by their conversion to pdbqt format. The grids were defined by choosing a co-crystallized ligand in the center of the box. The X, Y, and Z coordinates defining the center of the grid box for crystal structures 1DX6 and 5IKR are 3.627, 64.986, 64.364, and 38.042, 2.131, and 61.28, respectively. The size of the grid box is 25 Å from the center of the box in X, Y, and Z directions. Config files were generated to define the information of protein, ligands, and grid box. Molecular docking was performed using AutoDock Vina (AutoDock Vina, CA, USA) following our previous studies [20,55]. The results were generated in terms of binding affinity defined in kcal/mol. The top three binding modes were considered for analysis, and the binding mode, which displayed interactions with key residues, was considered for discussion.

### 4.13. Molecular Dynamics (MD) Simulations and MM/GBSA Calculations

The MD simulation of the COX-2 and AChE enzymes with docked ligand (ellagic acid) was performed using the Schrodinger Desmond 2021-1 software (New York, NY, USA) [56]. The Protein Preparation Wizard was used to prepare the complex proteins before running the MD simulation [57]. The initial protein–ligand complex was solved using the system builder using the transferable intermolecular potential 3P (TIP3P) water model in the orthorhombic box. A sufficient number of ions were added to the solvated complexes to neutralize the system, and 0.15 M NaCl salt was added to the complex to act as a buffer. These complexes were subsequently subjected to energy minimization for 100 ps. Following minimization, a 100 ns MD simulation of all compounds was run while taking into account the NPT ensemble, 300 K temperature, and 1.01325 bar pressure. Furthermore, systems were relaxed before the main simulation started. For other parameters, default settings were used. The MM/GBSA method implemented in the DESMOND package was used to determine relative binding free energy. For this purpose, 50 frames were extracted from the last 500 snapshots at an interval of 10 steps. Using the thermal mmgbsa.py script, ΔGbind energies were calculated based on the equation below.

ΔGbind = Gcomplex − Gprotein (unbound) − G ligand (unbound)

where, Gcomplex is the MM/GBSA energy of the minimized enzyme-ellagic acid complex, Gprotein (unbound) is the MM/GBSA energy of the relaxed enzyme from which ellagic acid was removed, and Glig(unbound) is the MM/GBSA energy of the ellagic acid in an unbound state where the ligand is separated from the complex and subjected to relaxation. In addition, the script also gives ΔGbind (NS) energies where enzyme and ligand relaxation energies are not considered in the computation of binding free energies. Finally, binding energies were decomposed to get deeper insight type of interactions contributing to binding energies.

## 5. Conclusions

Overall, from the results, it can be concluded that by using an LPS-induced experimental model, MASE might be a viable neuroprotective approach against cognitive impairment and neural inflammation. In both the EPM and NOR tests, MASE administration enhanced spatial learning and memory. The treatment also increased the level of ACh in the brain, which improved cholinergic neuronal activity. Furthermore, it displayed anti-inflammatory potential in the rat brain by reducing LPS-induced elevations of the COX-2 enzyme, pro-inflammatory cytokines (TNF-α, and IL-6) levels, and improved anti-inflammatory cytokines (TGF-β1 and IL-10) levels. Based on these findings, MASE may be a promising treatment for reducing neuroinflammatory damage from a number of neurodegenerative diseases. The phytochemical analysis reveals the presence of a significant amount of phenolics and a considerable amount of flavonoids in MASE. Molecular docking and dynamic studies revealed that compounds ellagic acid, epicatechin, catechin, cinnamic acid, hesperidin, hesperetin, kaempferol narengin, quercetin, rutin, and apigenin have the potential to inhibit AChE by interacting with residue critical for hydrolysis of acetylcholine and can be responsible for the improvement of cholinergic transmission. The docking and dynamic studies further revealed that ellagic acid, epicatechin, catechin, kaempferol, quercetin, and apigenin have the potential to block the hydrophobic channel of COX-2 and can be responsible for the improvement in inflammatory conditions. Furthermore, the study can be extended to explore other inflammatory parameters like chemokines, ROS, secondary messengers, and other cytokines.

## Figures and Tables

**Table 1:** Quantitative measurements of the total polyphenols, total flavonoids, and total tannin contents in Ajwa seed extract (MASE).

TPC	TFC	TTC
mg/gm of the dried plant extract		
76.06 ± 0.94	8.66 ± 0.02	11.48 ± 1.19

All the measurements were conducted in triplicate; the mean ± standard deviations were calculated. TPC—total phenolic contents calculated in mg/g gallic acid equivalent; TFC—total flavonoid contents calculated in mg/g quercetin equivalent; TTC—total tannins contents calculated in mg/gm catechin equivalent.

**Table 2:** The calculated binding affinity of phytochemicals from date seed.

Sr. No.	Ligands	AChE	COX-2
1	Co L	−10.5	−9.1
2	Ellagic acid	−10.2	−7.5
3	Epicatechin	−9.6	−7
4	Catechin	−9.3	−7.4
5	Pyragallol	−5.4	−5.4
6	Syringic acid	−6.3	−6
7	Vanillic acid	−6.1	−6.1
8	Benzoic acid	−6.4	−5.5
9	Catechol	−5.4	−5.2
10	Cinnamic acid	−7	−6.1
11	Gallic acid	−6.3	−5.9
12	Hesperidin	−11.2	−6.6
13	Hesperetin	−10.3	−5.5
14	Kaempferol	−9.9	−7.8
15	Narengin	−11.1	−3.5
16	Quercetin	−9.6	−7.9
17	Rutin	−10.9	−4.7
18	Apigenin	−9.9	−8.3

**Table 3:** Residue-wise interaction analysis for different ligands against AChE.

Sr. No.	Ligands	Residue-Wise Interaction (Hydrogen and Hydrophobic)
1	Ellagic acid	Asp72, Trp84, Gly117, Tyr130, Glu199, Phe330, His440,
2	Epicatechin	Trp84, Asn85, Tyr121, Ser122, Glu199,
3	Catechin	Trp84, Gly117, Gly118,Tyr121, Ser122, Tyr130, Phe330, Phe331,
4	Pyragallol	Trp84, Tyr130, Glu199,
5	Syringic acid	Asp72, Trp84, Phe330, Phe331, Tyr334, His440
6	Vanillic acid	Tyr121, Ser122, Glu199, Ser200, His440
7	Benzoic acid	Phe330, Phe331 Tyr334
8	Catechol	Asp72, Phe330, Phe331, Tyr334
9	Cinnamic acid	Gly119, Ser200, Phe330, Phe331, Tyr334, His440
10	Gallic acid	Trp84, Ser122, Tyr130, Glu199
11	Hesperidin	Tyr70, Asp72, Trp84, Asn85, Gly118, Trp279, Ile287, Phe330, Phe331, Tyr334, His440
12	Hesperetin	Asp72, Trp84, Tyr121, Ser122, Ile287, Phe330, Phe331
13	Kaempferol	Trp84, Tyr121, Ser122, Tyr130, Tyr334
14	Narengin	Trp84, Asn85, Gly118, Ser122, Glu199, Ser200, Trp279, Phe331, Tyr334, His440
15	Quercetin	Trp84, Gly117, Gly118, Tyr121, Ser122, Tyr130, Phe330,
16	Rutin	Tyr70, Tyr121, Ser122, Ser286, Arg289, Trp279, Phe290
17	Apigenin	Tyr70, Trp84, Asn85, Tyr121, Ser122

**Table 4:** Residue-wise interaction analysis for different ligands against COX-2.

Sr. No.	Ligands	Residue-Wise Interaction (Hydrogen and Hydrophobic)
1	Ellagic acid	Ser530, Val349, Leu352, Ala527, Gly526, Val523
2	Epicatechin	Val116, Val349, Leu352, Tyr355, Leu359, Tyr385, Gly526, Val523, Ala527, Leu531
3	Catechin	Val116, Arg120, Val349, Leu352, Ala527, Leu531
4	Pyragallol	Leu352, Val523, Ala527
5	Syringic acid	Val349, Tyr385, Trp387, Leu352, Phe518, Met522, Val523, Ala527, Leu531
6	Vanillic acid	Val349, Leu352, Val523, Gly526, Ala527, Leu531,
7	Benzoic acid	Tyr385, Leu352, Phe518, Val523, Ala527 Ser530,
8	Catechol	Val349, Leu352, Met522, Val523, Ala527
9	Cinnamic acid	Arg120, Val349, Leu352, Ser353, Tyr355, Ala527
10	Gallic acid	Leu352, Tyr385, Gly526, Ala527 Ser530,
11	Hesperidin	Tyr115, Val116, Ser119, Arg120, Tyr355, Phe381, Leu384, Tyr385, Trp387, Val349, Leu352, Ala527, Met522, Val523, Ala527, Leu531
12	Hesperetin	Val116, Arg120, Val349, Leu352, Leu359, Tyr385, Trp387, Ala527, Ala527, Leu531
13	Kaempferol	Arg120, Val349, Leu352, Phe518, Val523, Gly526, Ala527, Ser530, Leu531,
14	Narengin	VAL89, VAL116, ARG120, VAL349, LEU352, TYR355, VAL523, GLU524, GLY526, ALA527
15	Quercetin	Val349, Leu352, Phe518, Val523, Gly526, Ala527, Ser530, Leu531, Ala527,
16	Rutin	Pro86, Val89, Leu93, Met113, Val116, Arg120, Val349, Leu352, Tyr355, Leu359, Tyr385, Ala527, Leu531
17	Apigenin	Val116, Arg120, Val349, Leu352, Tyr355, Ala527, Leu531, Leu359, Ala527

## Data Availability

The data presented in this study are available from the corresponding author upon reasonable request.

## Sample Availability

Samples of the plant extract are available from the authors.
